# Glenaings from My Journal of Five, Years Practice.—On Exposed Nerves

**Published:** 1855-07

**Authors:** J. Sanford

**Affiliations:** Chillicothe, O.


### GLENAINGS FROM MY JOURNAL OF FIVE, YEARS PRACTICE.—ON EXPOSED NERVES.

As the question of filling teeth, over exposed nerves, seems to be exciting more than usual interest at this time, I feel it a duty incumbent on me to contribute my *Journal Entries*, not that I expect to enlighten the whole dental profession, but probably it may be of service to the young and inexperienced practitioner, of our worthy calling. For the last five years I have used spirits of camphor in allaying sensitiveness of the nerves in teeth, I speak here from actual practice and record. I have noted down in my Journal thirty cases, and every case where the nerves have been exposed, and in a healthy state, I have filled and saved the teeth. I will now give my mode of operating. In the first place I excavate the cavity and when caries have went so far as to approach the dental pulp I apply the spirits of camphor on a little raw cotton to the exposed nerve which in nine cases out of ten will arrest the hemorrhage, in one or two applications; when arrested, I continue excavating, until I have a good cavity. After the cavity is well cleansed, I then apply the camphor, three or four times, in as many minutes and you are ready to introduce the gold. In this part of the operation it is very important the foil should be condensed very firmly, and finished well. I will here state, that after introducing, your camphor to the exposed nerve, as above directed it will produce no pain on introducing the foil immediately over the exposed nerve, this is a good test to determine when you have applied the remedy sufficiently.

The teeth I have been so fortunate with, have been incisors and canine of superior maxilla ; I have also experimented on bicuspids and molars which are doing as well as the first named, and I am confident will act well; they have not been on test so long as the first named. The beauty of this operation is, in the teeth retaining their natural beautiful transparency, and vitality. It seems that by the powerful astringent properties of the camphor, the nerve is contracted within its cavity and if well filled heals from first intention. I have tried arching, capping, and risodontropy on exposed nerves and I must confess I have not succeeded so satisfacorily as many other operators. The remedy is applied in no case but healthy exposed nerves.

CHILLICOTHE, O. J. SANFORD.

